# Optimal follow-up period after switching to another inhaled corticosteroid/long-acting β2 agonist in patients with asthma: A retrospective study using Japanese administrative claims data

**DOI:** 10.1371/journal.pone.0276001

**Published:** 2022-10-13

**Authors:** Rieko Kondo, Shotaro Maeda, Akira Kikuchi, Hiromichi Kiyono, Tohru Sato

**Affiliations:** 1 Kondo Clinic of Internal Medicine, Hamamatsu, Shizuoka, Japan; 2 Medical Affairs, Kyorin Pharmaceutical Co., Ltd., Tokyo, Japan; University Putra Malaysia, MALAYSIA

## Abstract

Switching inhalation devices is a reasonable option if problems with control, adherence, or inhalation technique occur in patients with asthma treated with inhaled corticosteroid (ICS)/long-acting β2 agonist (LABA). However, evidence to determine the extent to which the carefully monitored period persists after switching is insufficient. In this study, we aimed to investigate the duration of the carefully monitored period after switching to another ICS/LABA. This retrospective study used claims data from Japanese health insurance associations from May 2014 to April 2019. A total of 1,951 patients who switched to another ICS/LABA during the study period were selected for analysis. The relative risk of the first exacerbation after switching was calculated for each four-week interval after the switch compared with that before the switch in a self-controlled case series design. We further assessed patient background associated with exacerbations during the follow-up period. In the primary analysis, the risk of asthma exacerbation compared to the control period was derived from a conditional logistic regression model, which showed a significant decrease immediately after the switch (1 to 4 weeks, Odds ratio [OR] 0.37, 95% confidence interval [CI] 0.26–0.54). Subsequently, the risk increased again and was not significantly different from the control period until week 32 (OR 0.55, 95% CI 0.29–1.04). In a sensitivity analysis among patients with a history of exacerbations, up to week 20 was the period of no continuous risk reduction (OR 0.84, 95% CI 0.41–1.70). In the secondary analysis, chronic rhinosinusitis, sleep disorders, and a history of asthma exacerbation were significantly associated with asthma exacerbation. The incidence of exacerbation remained high for approximately 4 to 7 months after patients with asthma switched to another ICS/LABA. Therefore, these patients should be carefully monitored for at least 4 to 7 months and should be re-assessed at an earlier point in time, if necessary.

## Introduction

Asthma is defined by Japanese guidelines as a disease characterized by clinical symptoms such as wheezing, dyspnea, chest tightness, and cough due to airway obstruction caused by chronic inflammation of the airways. The goal of asthma treatment is to control airway inflammation and achieve sufficient airway dilation by avoiding and removing the risk factors that cause airway inflammation and by providing appropriate treatment [1]. Inhaled corticosteroid (ICS)/long-acting β2 agonist (LABA) has been recognized as the gold standard for the treatment of asthma [2, 3]. It is recommended in the early stage of treatment as per the Japanese guidelines [1]. Adachi et al. reported a prescription rate of 99% for ICS or ICS/LABA in a survey conducted on respiratory and allergy departments [4]. Hozawa et al. reported a prescription rate of 60% across all departments, indicating that ICS/LABA is widely used in clinical practice [5].

Although ICS/LABA has been widely used as the gold standard therapy, inadequate adherence and inhalation technique have been reported in many patients in actual clinical practice [6–8]; thus, inadequate asthma control remains a problem. When the response to treatment is poor, a change in the device (hereinafter referred to as “switch”) is recommended after assessment of the inhalation technique, and adherence and good control are not achieved, even following repeated inhalation instructions [1]. In addition, other Japanese practical guidelines (Practical Guidelines for Asthma Management 2021) recommend a treatable trait approach that considers the individual characteristics of each patient to determine a treatment strategy for patients who do not respond well to treatment [9]. Switching ICS/LABA is a reasonable option for reassessing the appropriate device for each patient because corticosteroid receptor activation differs depending on the combination pattern of each ICS and LABA [10, 11], and the inspiratory flow rate, handling technique, and the number of inhalations required vary depending on the device (dry powder inhaler or pressurized metered-dose inhaler) [12–16].

While switching is a reasonable treatment option, good control may not possibly be achieved if it is not performed adequately [17]. Therefore, careful follow-up after switching is required; however, it is not clear when the carefully monitored period is when exacerbations are most likely to occur and how long it lasts. In addition, the Japanese guidelines also suggest re-assessment every 3–6 months during the maintenance phase of treatment [1], but specific reports on the basis of this period are not available.

A better understanding of this information will enable a better post-switch follow-up. Therefore, the primary objective of this study was to evaluate the duration of the carefully monitored period for exacerbation occurrence after patients with asthma switched to another ICS/LABA, and the secondary objective was to investigate patient background associated with exacerbations.

## Materials and methods

### Study design and data source

**Fig 1:**
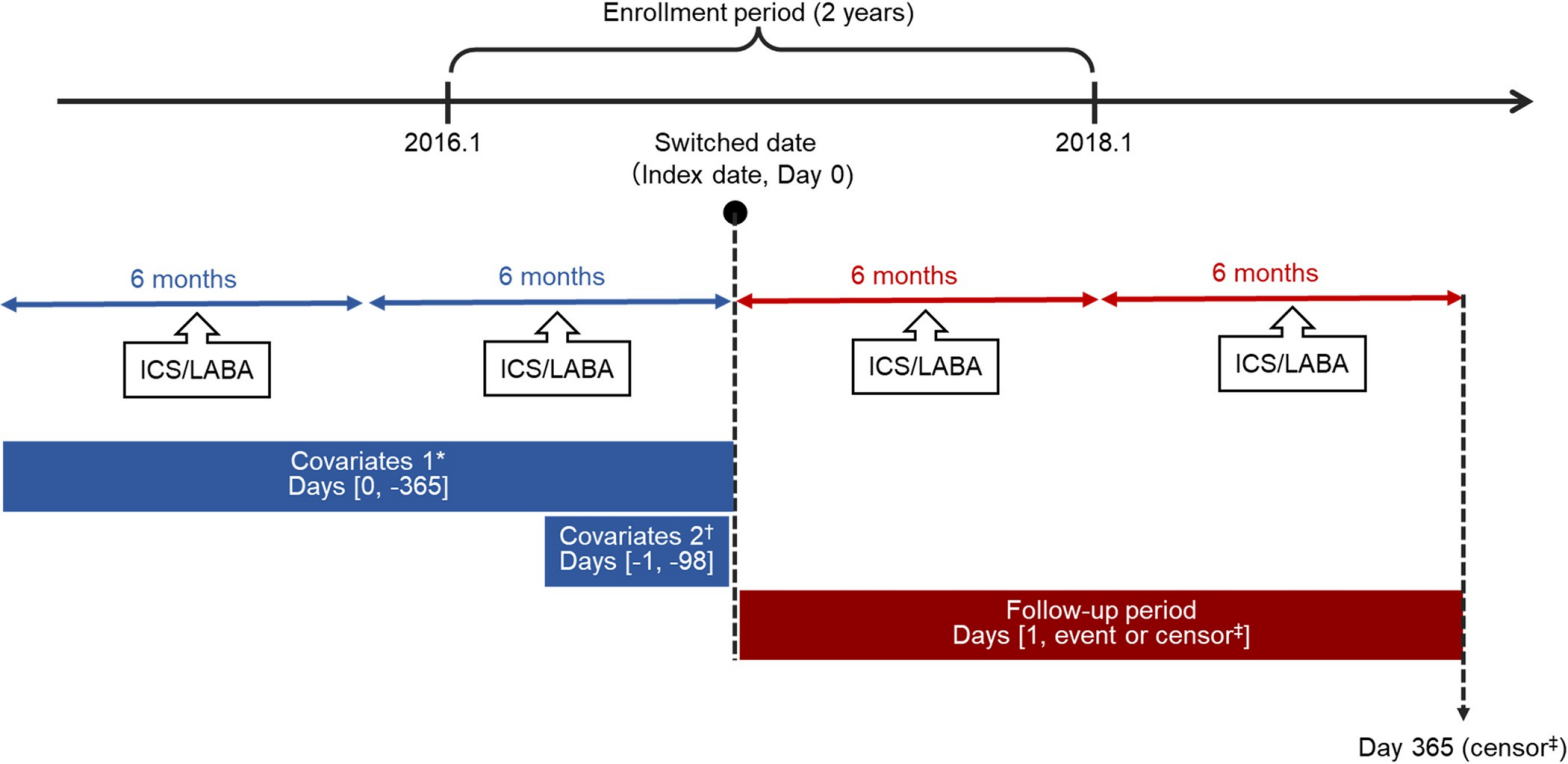
Study design. ICS, inhaled corticosteroid; LABA, long-acting β2-agonist. * Medical history and complications, respiratory function-related tests, asthma treatment management fee, and history of asthma exacerbations. † Asthma treatments (ICS, leukotriene receptor antagonist, slow-release theophylline, disodium cromoglycate, long-acting muscarinic antagonist, LABA, oral corticosteroid, and biologic agents). ‡ Censoring by re-switching to another ICS/LABA, adding an ICS, or switching to an ICS.

This retrospective study used data from MediScope® (INTAGE Real World Inc.), a Japanese claims database. The data consist of medical, dental, dispensing, and diagnosis procedure combinations collected from health insurance associations in Japan. The data also include age, sex, diagnosed disease, medication, and medical treatment of the patients, as well as the region of the patients and their families. A list of databases that can be used for research in Japan, including MediScope®, can be found on the website of the Japanese Society for Pharmacoepidemiology [18]. In this study, we used a dataset of patients who had asthma diagnoses (N = 1,748,111) according to the International Classification of Diseases, 10^th^ revision (ICD-10) codes J45 (asthma) or J46 (asthma attack severity) between May 2014 and April 2019. The design of the study and time window are shown in Fig 1.

### Study participants

**Fig 2:**
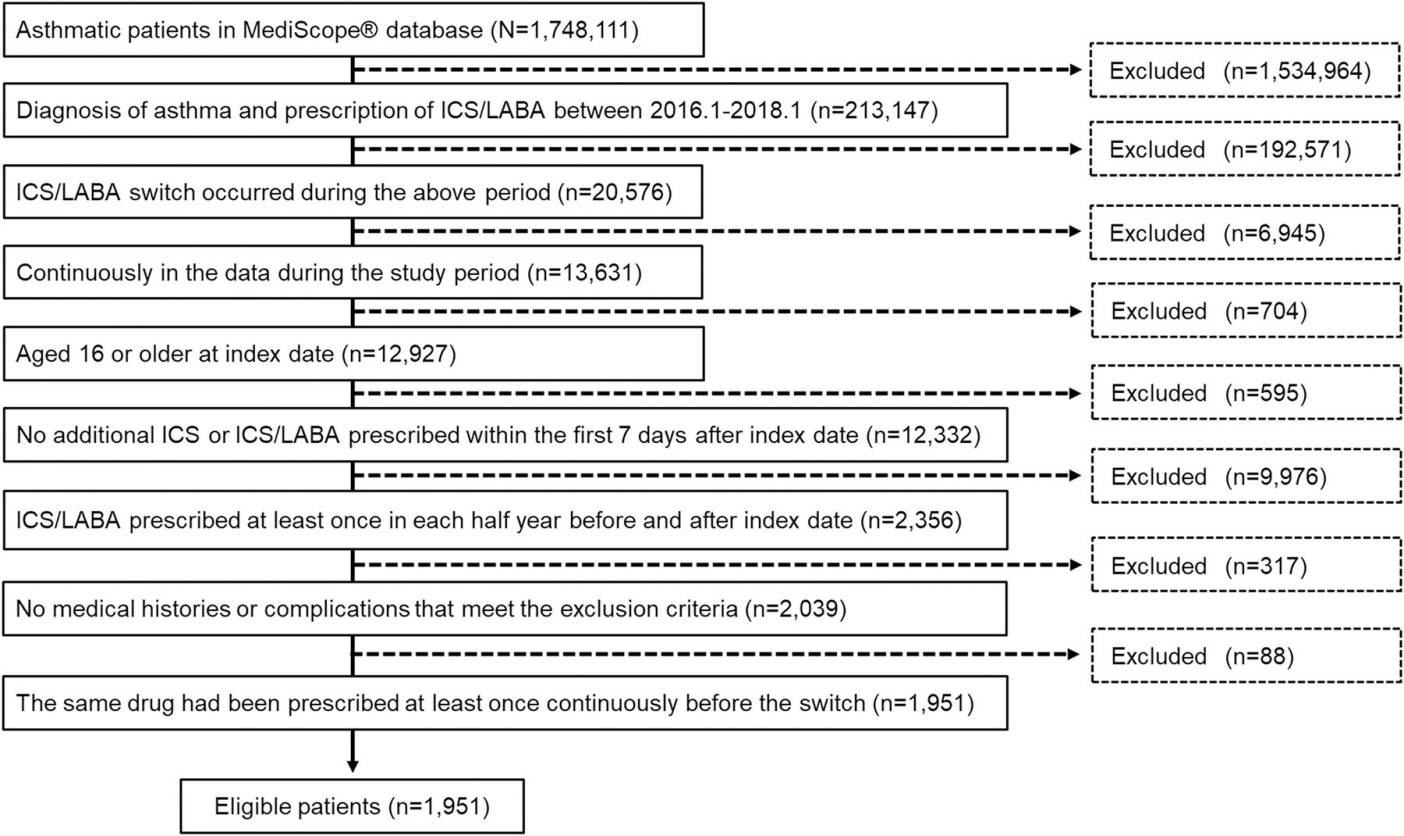
Flow of eligible patients. ICS, inhaled corticosteroid; LABA, long-acting β2-agonist.

Patients who met all the criteria, shown in Fig 2, were included in the analysis. Diseases excluded from the analysis are listed in the S1 Table.

### Outcomes

The outcomes of this study were asthma exacerbations, which were defined using the ICD-10 codes for diagnoses, Anatomical Therapeutic Chemical Classification System codes, generic names for prescriptions, and Japan-specific standardized procedures for medical treatment [19]. Asthma exacerbation was defined as the occurrence of the composite endpoint of emergency medical care, hospitalization, systemic corticosteroid/aminophylline administration, short-acting beta-2 agonist (SABA) inhalation at a medical institution, and a short burst of oral corticosteroid (OCS) during the follow-up period. The definition of each event is presented in the S2 Table. The number of days between the index date and the onset of exacerbations was calculated for the latter analyses.

### Variables

Age and sex at the index date, asthma treatment within 3 months prior to the index date, and records of medical history, comorbidities, and respiratory tests within 1 year prior to the index date were extracted as variables. Since a history of previous exacerbations is considered to be closely related to exacerbations during the follow-up period [20, 21], we calculated the “exacerbation-free period” as the time until the exacerbation that occurred prior to the index date. For medical history and comorbidities, the factors that were reported to be associated with risk in the guidelines and could be defined in this database were selected [1, 9]. For asthma treatment, prescription records of biologic agents, disodium cromoglycate, ICS, LABA, long-acting muscarinic antagonist, leukotriene receptor antagonist, OCS, SABA, and slow-release theophylline approximately 3 months (98 days) prior to the index date and at the index date were extracted. Of these, “with prescription” was defined as when a record of prescription had occurred for 28 days or more, except for biologic agents and OCSs. Biologic agents were considered “with prescription” if they were prescribed at least once, and OCSs were considered “with prescription” if they were prescribed for more than 7 days, excluding short bursts. The treatment steps were calculated using drug combinations based on the criteria of the modified Japanese guidelines [1]. Definitions of the classification criteria are presented in the S3 Table. We also calculated the changes in the treatment steps for the latter analysis.

### Statistical analyses

For descriptive statistics, means (standard deviations) or medians (interquartile range) were calculated for continuous variables, and frequencies and proportions (%) were calculated for categorical variables. For each background, a chi-square test was conducted between the groups. For all tests, the significance level was two-tailed and set at 0.05.

In the primary analysis, a self-controlled case series (SCCS) analysis was performed to examine the carefully monitored period regarding the first exacerbation after the index date. SCCS is a method for relative incidence [22], and “the key question is not ‘who’ but ‘when’.” [23]. It is a method used to estimate risk by dividing the patient’s period into case and control. The control period was defined as the period from the index date to 28 days prior to the index date. Subsequently, for the case period, the follow-up period was divided into 28-day intervals (creating 13 intervals), and the odds ratio (OR) between the case and control periods was estimated using a conditional logistic regression model. Although SCCS enables control of the confounding effect of patient background by self-control design, the switched season, the prescription of SABA, and the days elapsed since the last exacerbation remains a major confounder, therefore, we estimated the risk adjusted for these factors. The summary statistics for the variables included in the model are shown in S4 Table. The seasons were defined as spring (March to May), summer (June to August), autumn (September to November), and winter (December to February) based on a previous study of SCCS in Japanese patients with asthma [24]. For SABA, with or without prescription was included in each period, and for exacerbation history, the number of days since the last exacerbation was included in each period. As a sensitivity analysis of the primary analysis, a subgroup analysis was performed for patients with exacerbations before the index date. In addition, a sensitivity analyses were performed for the outcome as breakthrough exacerbation (Short-burst of OCS and SABA inhalation at the medical institution).

In the secondary analysis, we used a Cox proportional hazard model to estimate the hazard ratio (HR) and its 95% confidence interval (CI). Sex, medical histories/comorbidities, history of asthma exacerbation, prescription of SABA, change in the asthma treatment step at the time of switching, the performance of respiratory function tests, and switched medications were entered into the model as patient background for evaluation, and estimates were calculated with age, region, and month of switching as covariates.

R (version 4.12, R Core Team [2021]. R: A language and environment for statistical computing. R Foundation for Statistical Computing, Vienna, Austria. URL: https://www.R-project.org/) was used for all statistical analyses. The results of the study were reported in accordance with the recommendations of the Strengthening the Reporting of Observational Studies in Epidemiology (STROBE) [25].

### Ethics

As this study used only anonymized data, approval of a research ethics committee and informed consent were not required [26].

## Results

### Patient characteristics

Patient characteristics were categorized into exacerbation and non-exacerbation groups according to the occurrence of exacerbations during the follow-up period. Of the 1,951 patients included in the analysis, exacerbations occurred in 448 (23.0%). For each event, emergency care was provided to 16 patients (0.8%); hospitalization, 58 patients (3.0%); steroid/aminophylline injection administration, 203 patients (10.4%); SABA inhalation in medical institutions, 133 patients (6.8%); short bursts of oral steroids, 224 patients (11.5%) (including duplicates). The frequencies and proportions for sex, region, type of respiratory tests, asthma treatment management fee, the switched season, the asthma treatment step, comorbidity, medical history, asthma treatment, ICS dose before and after the switch, change of treatment (step up/down of asthma treatment), history of previous exacerbations, and the prescription of SABA are shown in Table 1.

**Table 1:** Baseline characteristics of patients. *P-values were derived using the chi-square test for categorical variables and the Wilcoxon rank sum test for continuous values.

	Total	Exacerbation group				*P*-value*
	(N = 1951)	No (n = 1503)		Yes (n = 448)		
**Age, years**						
Median (IQR)	47	47	(39–54)	47	(39–55)	0.978
**Sex, n (%)**						
Female	1171	875	74.7%	296	25.3%	0.003
Male	780	628	80.5%	152	19.5%	
**Area, n (%)**						
Hokkaido	105	76	72.4%	29	27.6%	0.154
Tohoku	154	113	73.4%	41	26.6%	
Kitakanto/Koshin	146	121	82.9%	25	17.1%	
Minamikanto	664	520	78.3%	144	21.7%	
Hokuriku	93	74	79.6%	19	20.4%	
Tokai	160	116	72.5%	44	27.5%	
Kinki	252	204	81.0%	48	19.0%	
Chugoku	94	68	72.3%	26	27.7%	
Shikoku	46	32	69.6%	14	30.4%	
Kyushu	237	179	75.5%	58	24.5%	
**Type of respiratory tests, n (%)**						
Spirometry						
Yes	512	374	73.0%	138	27.0%	0.132
No	1439	1129	78.5%	310	21.5%	
Forced Oscillation technique						
Yes	91	65	71.4%	26	28.6%	0.068
No	1860	1438	77.3%	422	22.7%	
FeNO						
Yes	342	245	71.6%	97	28.4%	0.137
No	1609	1258	78.2%	351	21.8%	
Any of the above tests						
Yes	670	499	74.5%	171	25.5%	0.104
No	1281	1004	78.4%	277	21.6%	
**Asthma treatment management fee, n (%)**						
Yes	88	62	70.5%	26	29.5%	0.133
No	1863	1441	77.3%	422	22.7%	
**Switched season, n (%)**						
Spring	529	409	77.3	120	22.7	0.484
Summer	535	423	79.1	112	20.9	
Autumn	157	121	77.1	36	22.9	
Winter	730	550	75.3	180	24.7	
**Medical history/Comorbidity, n (%)**						
Allergic rhinitis						
Yes	1466	1110	75.7%	356	24.3%	0.016
No	485	393	81.0%	92	19.0%	
Chronic sinusitis						
Yes	403	286	71.0%	117	29.0%	0.001
No	1548	1217	78.6%	331	21.4%	
GERD						
Yes	450	333	74.0%	117	26.0%	0.807
No	1501	1170	77.9%	331	22.1%	
Periodontitis						
Yes	912	703	77.1%	209	22.9%	0.964
No	1039	800	77.0%	239	23.0%	
Sleep disorder						
Yes	301	214	71.1%	87	28.9%	0.008
No	1650	1289	78.1%	361	21.9%	
Dyslipidemia						
Yes	471	364	77.3%	107	22.7%	0.885
No	1480	1139	77.0%	341	23.0%	
Anxiety/Depression						
Yes	207	158	76.3%	49	23.7%	0.798
No	1744	1345	77.1%	399	22.9%	
**History of exacerbation, n (%)**						
Within 30 days before Index date	237	96	40.5%	141	59.5%	< 0.001
31 to 91 days before Index date	98	47	48.0%	51	52.0%	
92 to 365 days before Index date	190	118	62.1%	72	37.9%	
No exacerbation	1426	1242	87.1%	184	12.9%	
**Prescription of SABA, n (%)**						
Yes	240	174	72.5%	66	27.5%	0.085
No	1711	1329	77.7%	382	22.3%	
**ICS dose, median (IQR), mcg/day**						
Before index date	307.7	285.7	(150.0–466.7)	363.6	(180.5–684.0)	< 0.001
At index date	395.6	387.1	(203.6–623.4)	403.6	(230.8–774.2)	0.005
**Change of treatment step, n (%)**						
Step down	419	285	68.0%	134	32.0%	< 0.001
Step up	298	234	78.5%	64	21.5%	
No change	1234	984	79.7%	250	20.3%	

IQR, interquartile range; FeNO, fractional exhaled nitric oxide; GERD, gastroesophageal reflux disease

**Fig 3:**
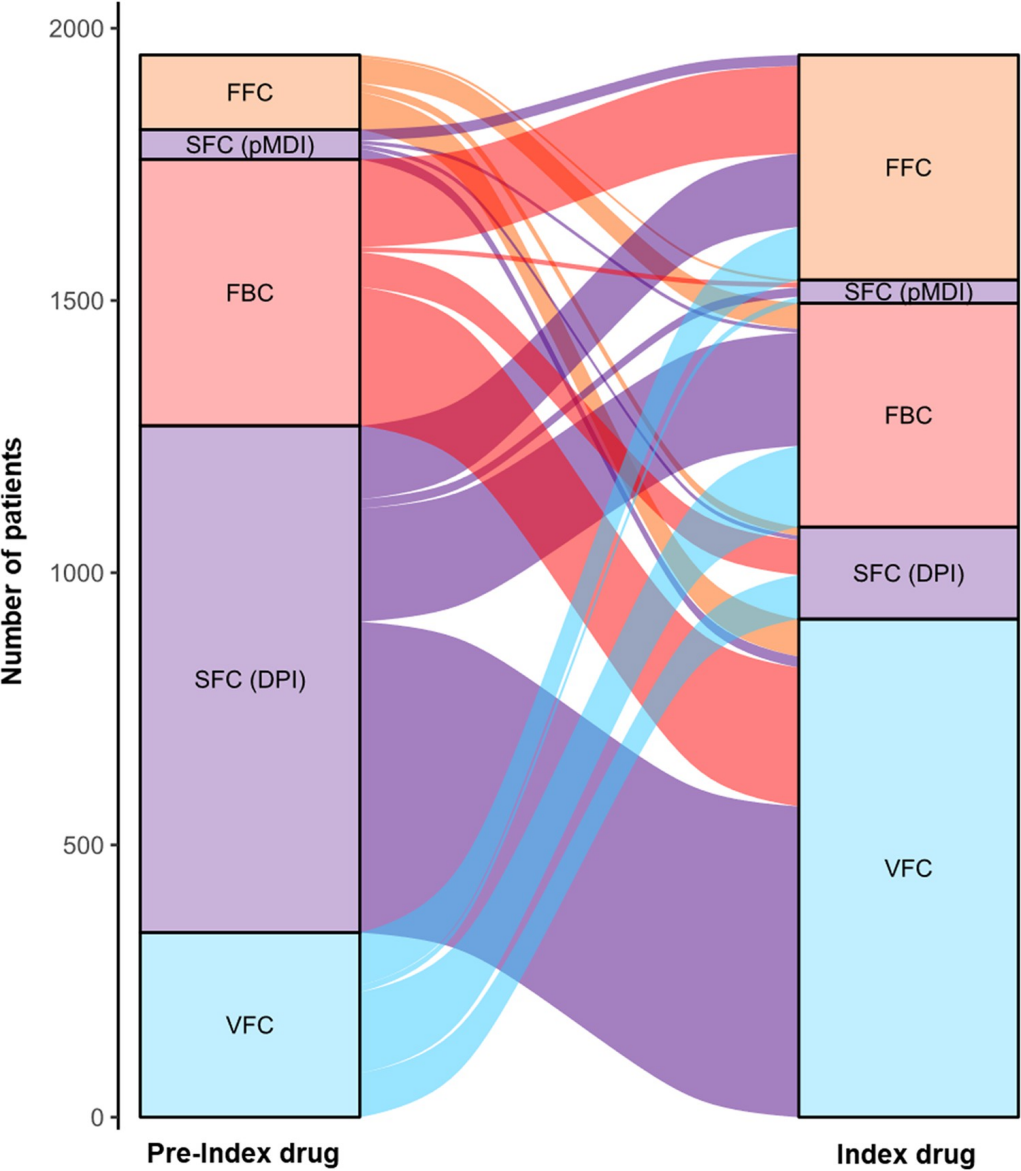
Switching patterns of ICS/LABA. FBC, formoterol/budesonide; FFC, formoterol/fluticasone propionate; SFC, salmeterol/fluticasone propionate; VFC, vilanterol/fluticasone furoate; DPI, dry powder inhaler; pMDI, pressurized metered-dose inhaler.

The switching pattern of the ICS/LABA is shown in the Sankey diagram in Fig 3.

### Primary and secondary analyses

**Fig 4:**
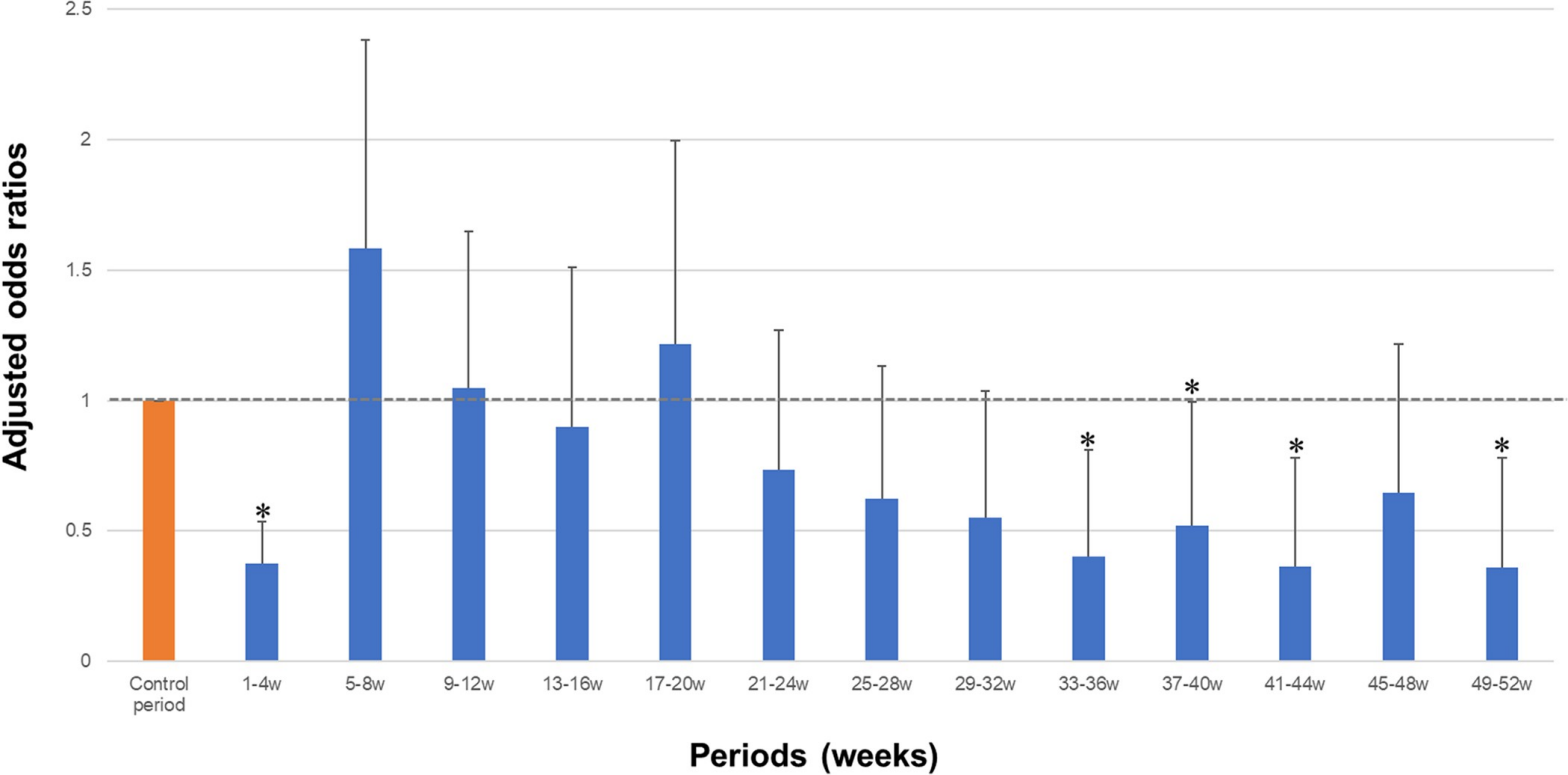
Odds ratios for each case period compared with the control period. Estimates and their 95% confidence intervals were derived from the self-controlled case series using conditional logistic regression. The model was adjusted for each season. * *P* < 0.05.

In the primary analysis, the ORs derived from SCCS are shown in Fig 4. The first significant decrease in the occurrence of asthma exacerbation events compared with the control period was observed at weeks 1–4 (OR 0.37, 95% CI 0.26–0.54). Subsequently, a significant decrease compared with the control period was observed at weeks 33–36, 37–40, 41–44, and 49–52 (OR 0.40, 95% CI 0.20–0.81, OR 0.52, 95% CI 0.27–0.99; OR 0.37, 95% CI 0.17–0.78; OR 0.36, 95% CI 0.17–0.78, respectively).

In the sensitivity analysis of the group who had an exacerbation before the index date, the risk decreased in 1–4 weeks immediately after the switch (OR 0.16, 95% CI 0.10–0.25) and did not differ significantly consecutively with the control period until week 20 (S1 Fig). For the sensitivity analyses of breakthrough exacerbations as an outcome, the risk decreased from 1 to 4 weeks immediately after the switch, and there was no continuous risk reduction up to week 24 in the entire population and in the population of patients with a history of exacerbations (S2 Fig).

**Fig 5:**
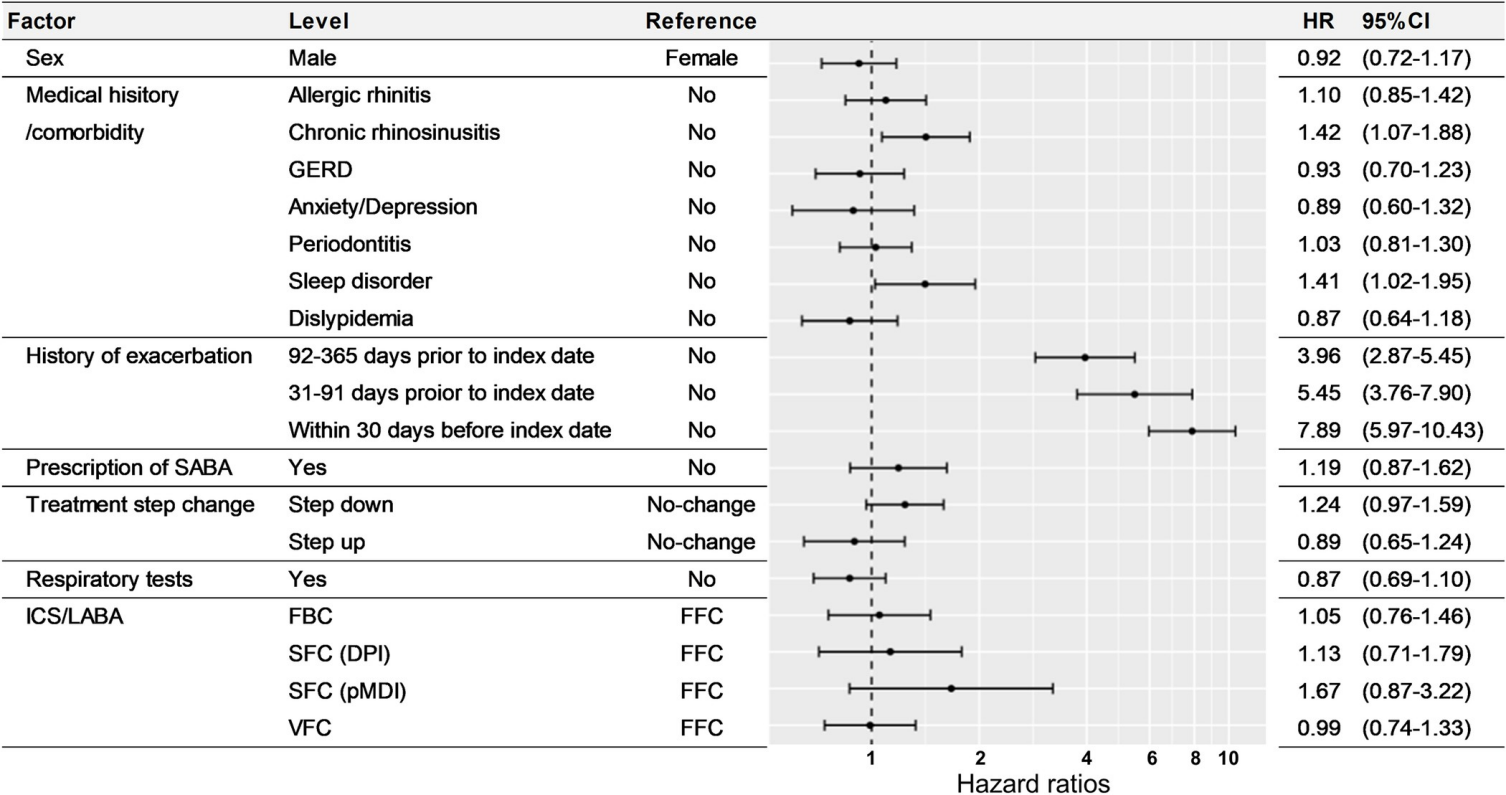
Factors associated with exacerbations. HR, hazard ratio; 95% CI, 95% confidence interval. HRs and 95% CIs were derived from multivariate Cox regression analysis adjusted for age, region, and month of switching, in addition to the variables listed above.

In the secondary analysis, the HR and its 95% CI for patients with chronic rhinosinusitis were 1.42 and 1.07–1.88; sleep disorder, 1.41 and 1.02–1.95; history of asthma exacerbation within 30 days before the index date, 7.89 and 5.97–10.43; between 31 and 91 days, 5.45 and 3.76–7.90; between 92 and 365 days, 3.96 and 2.87–5.45, respectively, which were significantly associated with asthma exacerbations (Fig 5).

## Discussion

In this study, the SCCS design was used to explore the carefully monitored period after switching to another ICS/LABA in patients treated with ICS/LABA. The results showed that the risk decreased immediately after the switch from 1 to 4 weeks, and then increased again, with no significant difference compared to the risk before the switch until the week 32. Chronic sinusitis, sleep disturbance, and a history of exacerbations before switching were statistically significant factors associated with exacerbations. To our knowledge, this study is the first to explore the carefully monitored period itself rather than the exacerbating factors in a specific period after switching among patients with asthma. The guidelines recommend re-assessment within 1 month after treatment initiation, and step down of treatment may be considered appropriate after 3 to 6 months [1].

The results of the primary analysis showed that the decrease in risk immediately after the ICS/LABA switch may have been influenced by inhalation technique guidance during the switch, improved adherence after the switch, and psychological effects of the switch. However, several factors are considered as possible reasons for the increased risk again in the subsequent period. First, the temporary effects of the ICS/LABA switch, such as improved inhalation technique and adherence, decreased. Second, the median date of the first visit after the switch was 29 days (S4 Table), and the opportunity to detect an event itself was relatively small during the initial period we defined (1–4 weeks). However, among the group with previous exacerbations in the sensitivity analysis, a period of no reduction in risk exists up to week 20 (approximately 4 months) post-switch, suggesting that these populations should be followed carefully in the closer post-switch period. Patients in this study include those for whom the reason for switching is unclear; however, based on the post-switch results for patients with a history of exacerbations who are likely to require switching, it is desirable to closely follow up on inhalation technique and adherence in addition to confirming response to treatment for about 4 months after the switch. However, even after the high-risk period, the risk remains unstable, indicating that regular follow-up is desirable for the carefully monitored period.

In this study, patients whose ICS/LABA was switched again after the switch were censored in the analysis. Therefore, patients who were assessed to be at risk were those who continued to use the same ICS/LABA during the study period. If control is insufficient after the switch, it should be reassessed at an early point, and ICS/LABA should be re-switched, or additional treatment should be considered. However, the fact that the carefully monitored period persisted for approximately 7 months (up to week 32) after the switch in the overall population, including patients with no history of exacerbations, suggests that the evaluation (follow-up) of the population after the switch was inadequate. Further studies on the timing, frequency, and options for appropriate evaluation are required.

In this study, chronic sinusitis, sleep disorder, and a history of exacerbation in the past year were significant risk factors, all of which were consistent with the results reported in previous studies [27–29]. Particularly for exacerbations in the past year, the risk was about eightfold higher in patients who had an exacerbation within 30 days than in those who had no exacerbations and was strongly associated with risk. A history of exacerbations prior to 91 days was also approximately 4.5 times higher than patients who had no exacerbations, suggesting that it is the most important factor to consider in patients who switched between ICS and LABA. In previous studies, allergic rhinitis [30], gastroesophageal reflux disease [31, 32], and periodontitis were associated with increased risk [33, 34], whereas dyslipidemia was associated with decreased risk [35, 36]. In this study, point estimates tended to be consistent with previous studies; however, they were not significantly associated with exacerbations. This difference may be owing to the difference in baseline risk severity and risk factors between general patients with asthma included in the previous study and those included in this study who required continuous ICS/LABA use and required a switch. Although no significant difference was found in the different drugs switched, the inhalation technique required for each ICS/LABA device was different, and if appropriate instructions were not given at the time of switching, the subsequent control may not be satisfactory [17]. In previous studies, the amount of drug reaching the lungs can differ significantly depending on the inhalation technique [37–39], and the difference is also considered to be substantial among ICS/LABA owing to differences in particle size and physical properties [40, 41]. Although the information on inhalation technique instruction was not available in the database or at this time, the pharmacy reimbursement system for inhalation instruction, which was started in 2020, will be recorded as claims data. Future analyses should consider the presence or absence of inhalation instruction.

The strengths of this study include the clinical relevance of analyzing a large database of more than 1.7 million individuals, reflecting actual clinical practice and, thus, providing results with high generalizability. Second, the use of the SCCS design with the patients themselves as controls provided results that were controlled for unmeasured confounding. These results may provide information that supports the description of duration in the guidelines.

This study has several limitations. First, the data used in this study were obtained from a specific health insurance association and are not representative of the Japanese population. In particular, the proportion of people aged 65 years or older was approximately 3.4%, which limits the generalizability of the results to the elderly. However, in terms of regional distribution, a comparison of the place of residence data published by the Statistics Bureau of the Ministry of Internal Affairs and Communications [42] and the place of the treatment of the participants in this study showed no prefectures with a standardized difference exceeding 0.1, except for three prefectures that were considered to represent the distribution in Japan (S5 Table).

Second, the data used in this study were submitted for insurance reimbursement, and the recorded disease names may have differed from the actual disease names. Therefore, there are limitations to the validity of the comorbidities assessed as variables and whether the outcomes actually occurred for asthma. Finally, inhalation instruction at the time of switching and subsequent adherence may be closely related to exacerbations; however, they could not be fully defined from this database, and the important factors may remain unmeasured confounders. In actual practice, there are various reasons for switching ICS/LABA, and instructions and confirmation of inhalation methods vary from physician to physician; nevertheless, it is important to carefully check the individual patient’s condition and consider the next treatment strategy [43]. Therefore, verifying the validity of the findings of this study in a clinical setting is warranted.

## Conclusions

The incidence of exacerbation events remained high for approximately up to 4 to 7 months after patients with asthma switched to another ICS/LABA. Therefore, patients who switched to another ICS/LABA are recommended to be followed up carefully for at least approximately up to 4 to 7 months. Reassessment should be performed at an earlier point in time if needed, and appropriate clinical intervention should be provided.

## Supporting information

S1 Fig Sensitivity analysis of the group who had an exacerbation before the index date. (TIF)

S2 Fig Sensitivity analyses of breakthrough exacerbations. (TIF)

S1 Table Diseases excluded from the analysis. (TIF)

S2 Table Definition of each exacerbation. (TIF)

S3 Table Definitions of treatment step classification. (TIF)

S4 Table Initial post-switch visits and SABA prescriptions in each period. (TIF)

S5 Table Differences in population distribution between MediScope® and the actual population in Japan. (TIF)

S6 Table ICS/LABA before and after switch. (TIF)

S7 Table Data for primary and sensitivity analysis. (TIF)
