# Supplementary figures and images for: Optimal follow-up period after switching to another inhaled corticosteroid/long-acting β2 agonist in patients with asthma: A retrospective study using Japanese administrative claims data
Source: PLoS One. 2022 Oct 13;17(10):e0276001. doi: 10.1371/journal.pone.0276001 (PMC9560144; doi:10.1371/journal.pone.0276001)

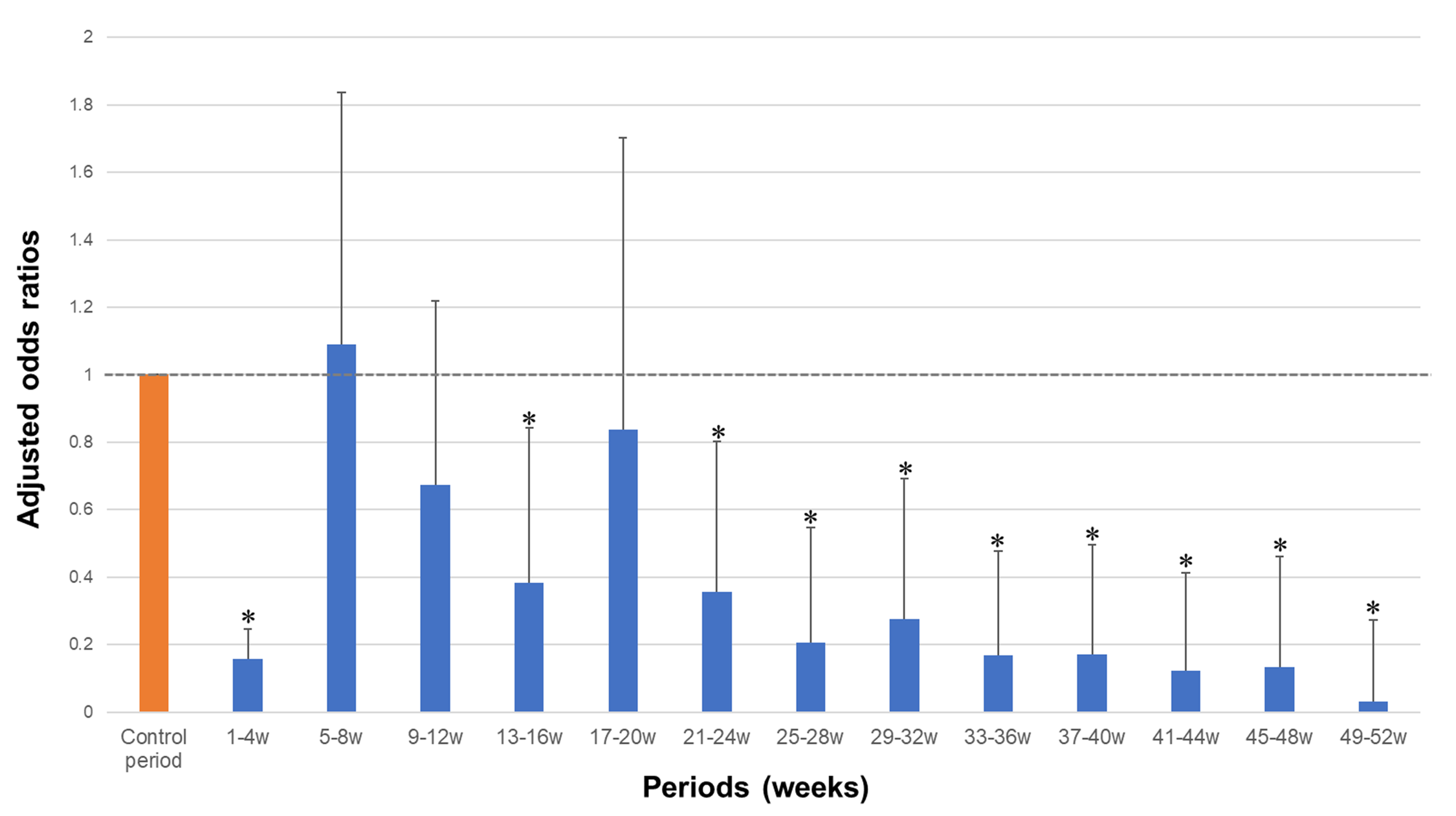

Supplement: S1 Fig — (TIF) [file pone.0276001.s001.tif]

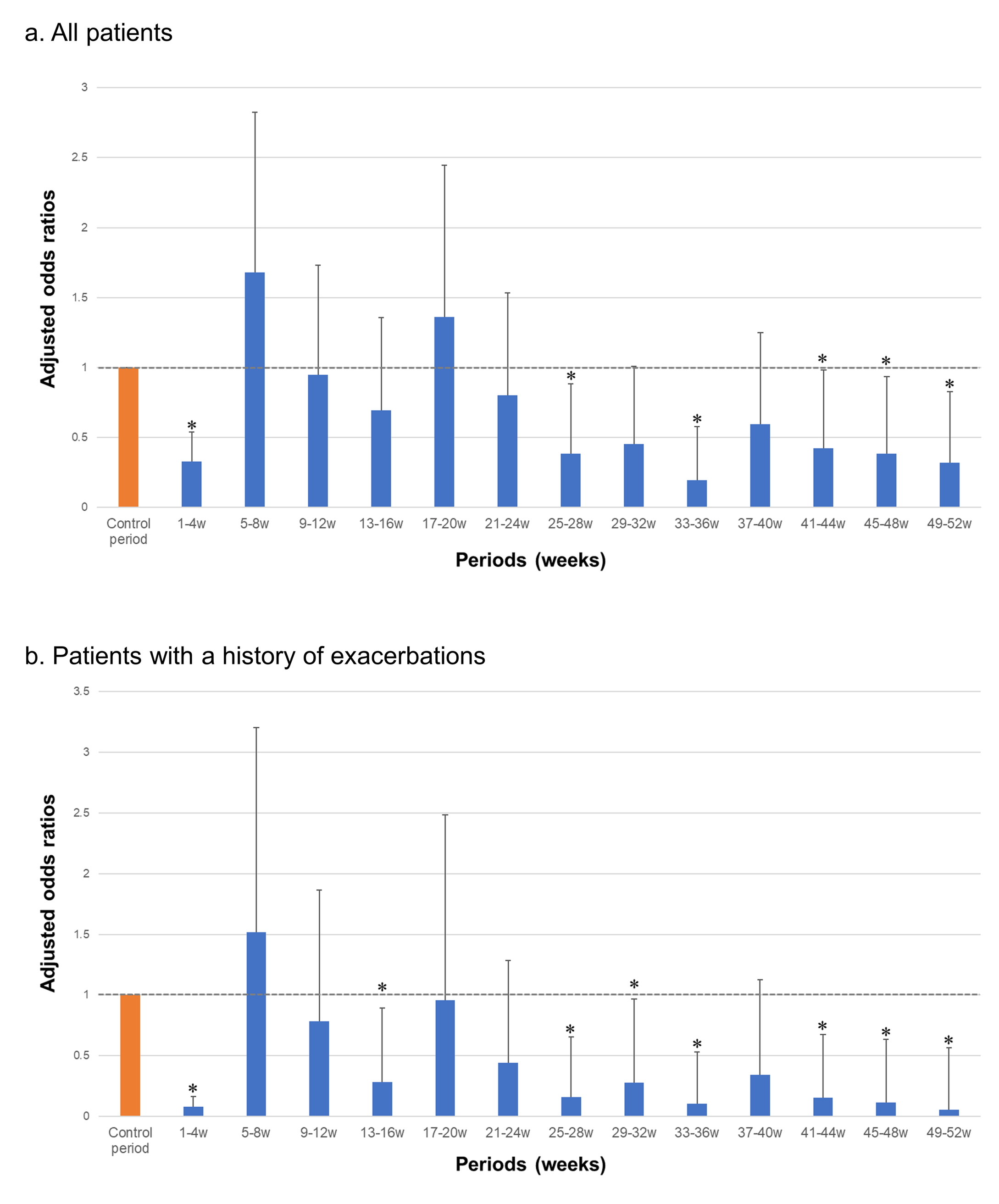

Supplement: S2 Fig — (TIF) [file pone.0276001.s002.tif]

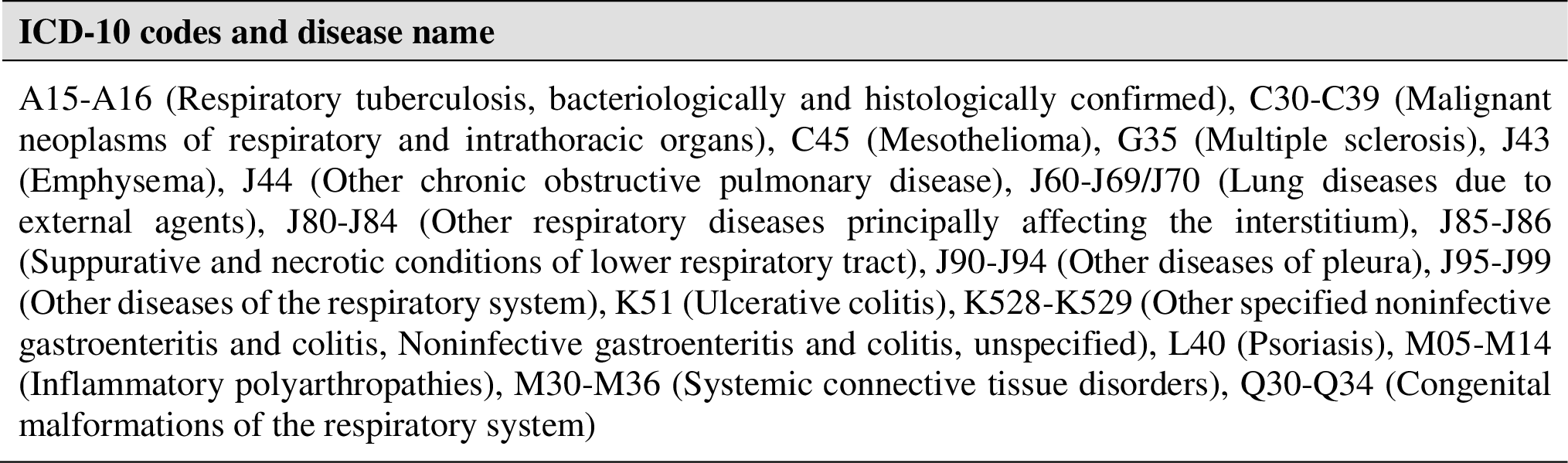

Supplement: S1 Table — (TIF) [file pone.0276001.s003.tif]

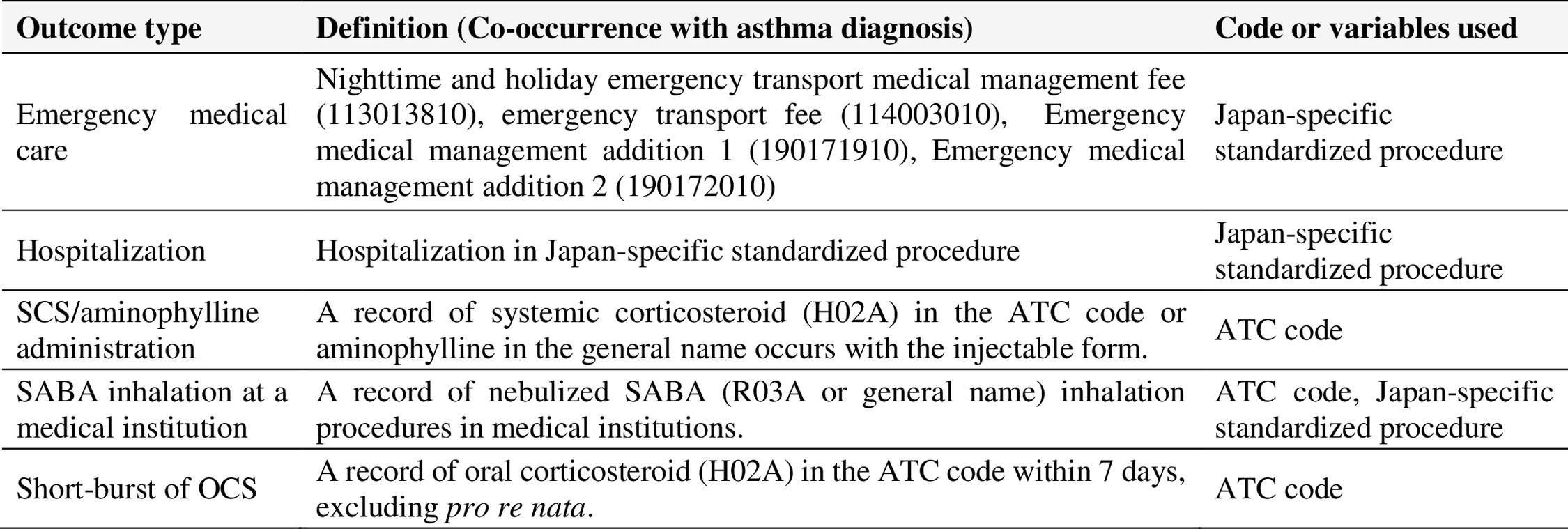

Supplement: S2 Table — (TIF) [file pone.0276001.s004.tif]

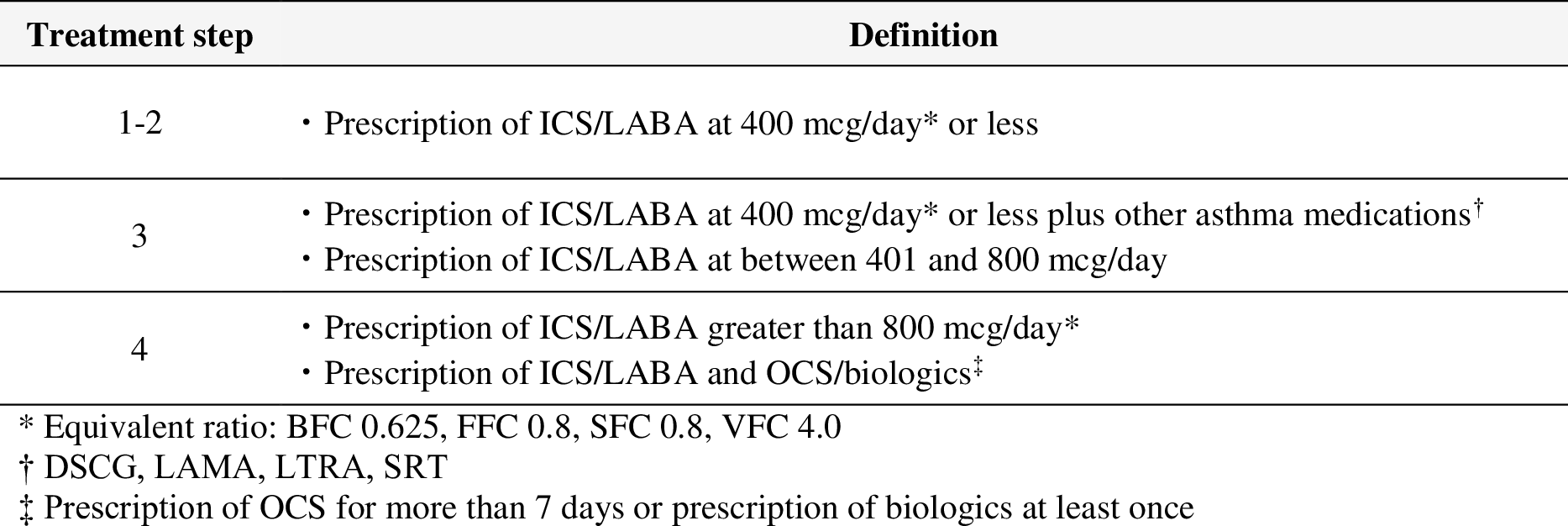

Supplement: S3 Table — (TIF) [file pone.0276001.s005.tif]

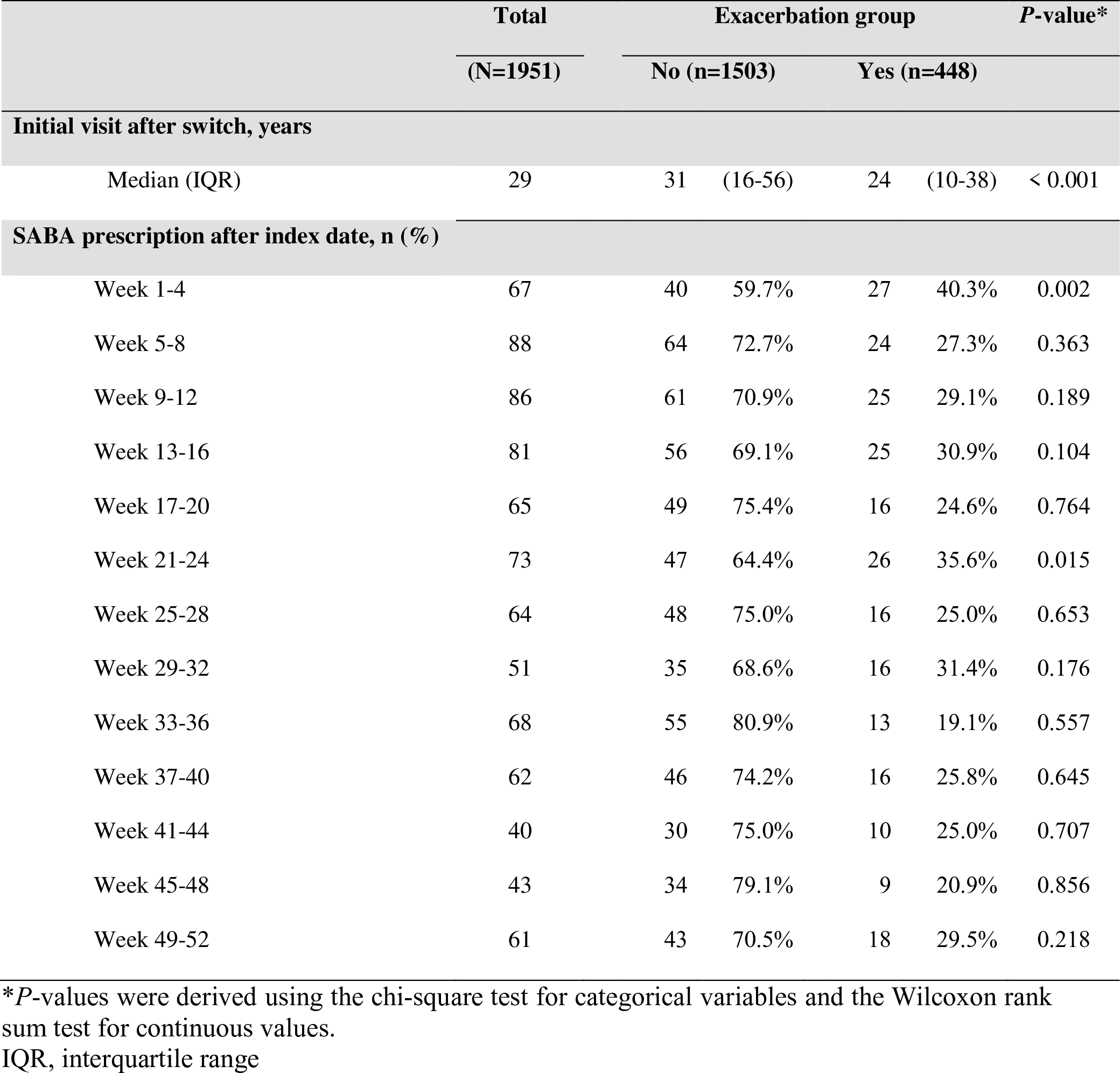

Supplement: S4 Table — (TIF) [file pone.0276001.s006.tif]

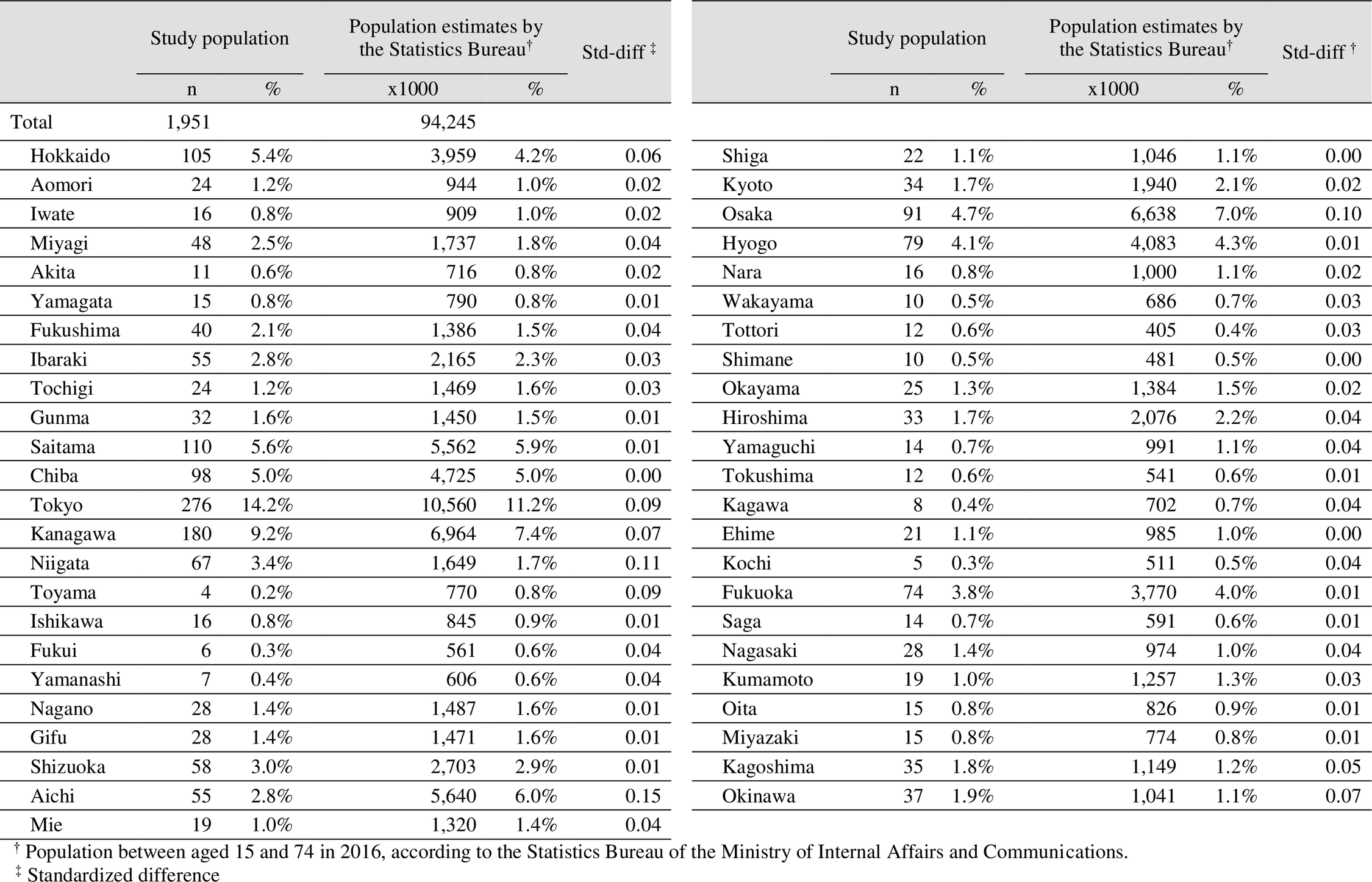

Supplement: S5 Table — (TIF) [file pone.0276001.s007.tif]

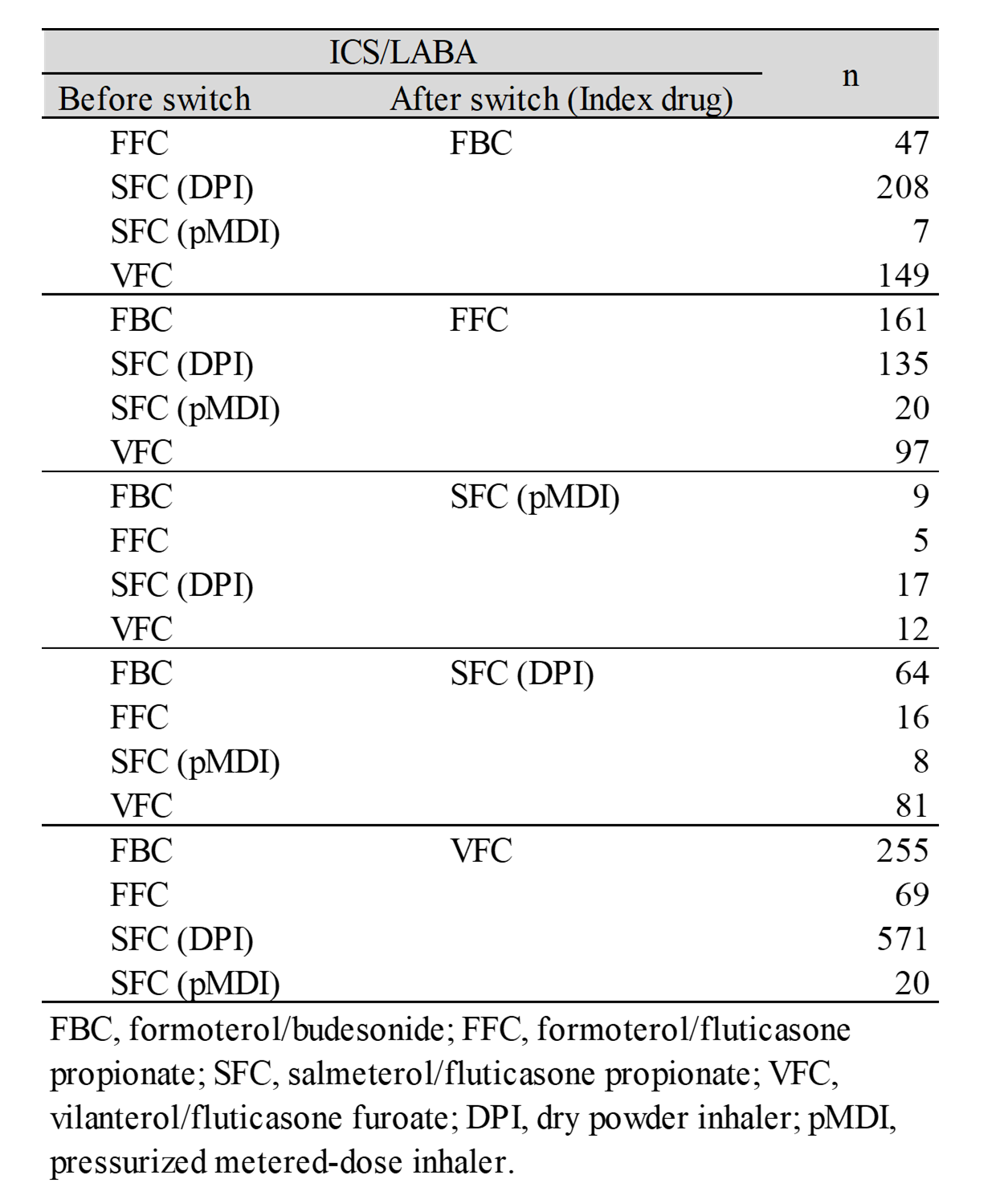

Supplement: S6 Table — (TIF) [file pone.0276001.s008.tif]

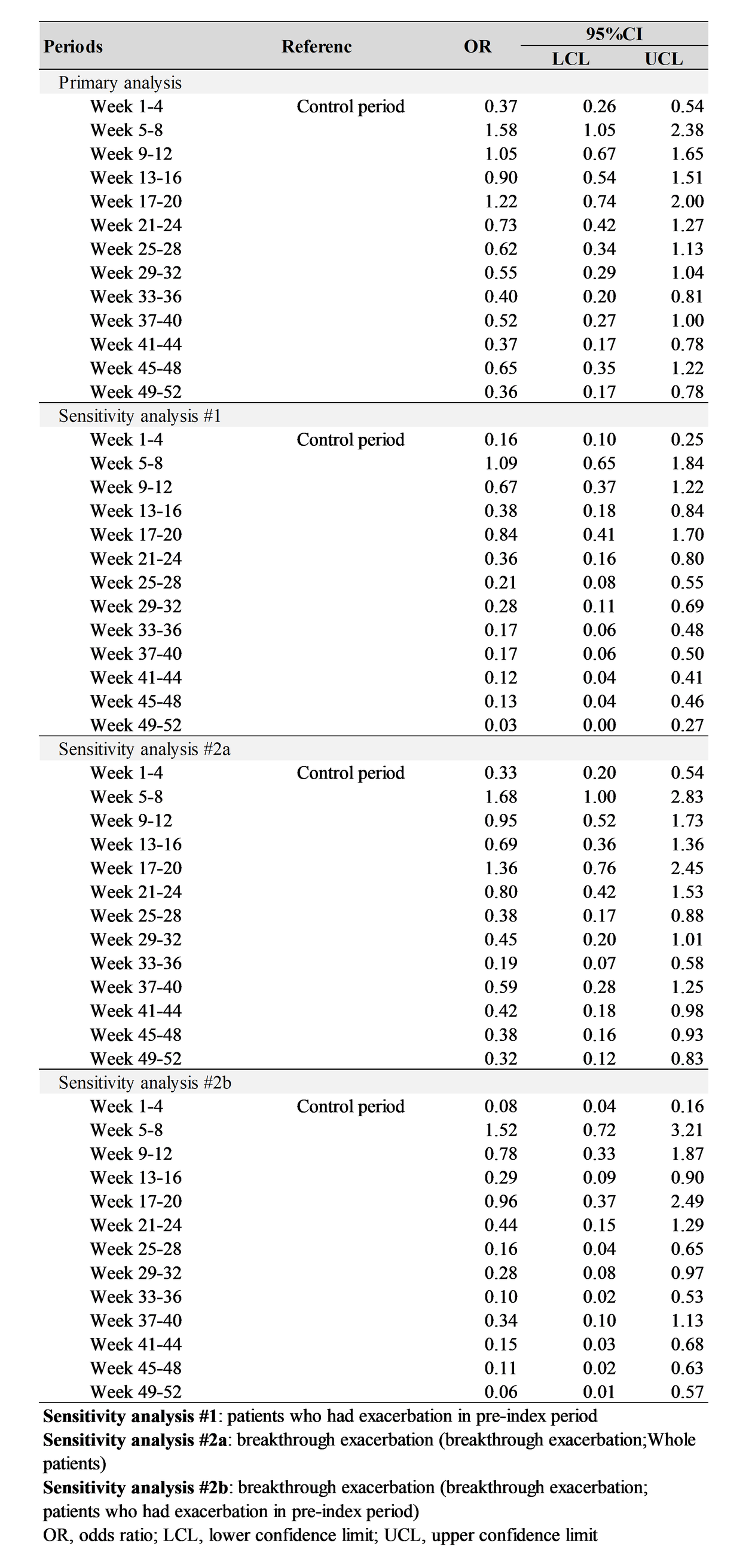

Supplement: S7 Table — (TIF) [file pone.0276001.s009.tif]
